# 1-[3-Meth­oxy-4-(prop-2-yn-1-yl­oxy)phen­yl]ethanone

**DOI:** 10.1107/S1600536810052074

**Published:** 2010-12-18

**Authors:** Chun-Hua Zhang, Jing-Min Zhao, Bao-Guo Chen

**Affiliations:** a College of Chemistry and Chemical Engineering, Inner Mongolia University for the Nationalities, Inner Mongolia Autonomous Region Tongliao, 22 Huolinhe street, 028000, People’s Republic of China; b Institute of Higher Vocational Education, Tongliao Vocational College, Inner Mongolia Autonomous Region Tongliao, 152 Huolinhe street, 028000, People’s Republic of China

## Abstract

In the title compound, C_12_H_12_O_3_, the meth­oxy and prop-2-yn­yloxy groups are nearly coplanar with the attached benzene ring [C—O—C—C torsion angles = 1.2 (3) and 2.2 (3)°, respectively]. In the crystal, inversion dimers linked by pairs of C—H⋯O inter­actions occur.

## Related literature

For the β-O-4 substructure in lignin, see: Cathala *et al.* (2003 ▶). For attempts to prepare well defined linear polymers with the β-O-4 structure and to develop new methods of utilizing lignins, see: Kishimoto *et al.* (2005 ▶). For a related structure, see: Yang *et al.* (2009 ▶).
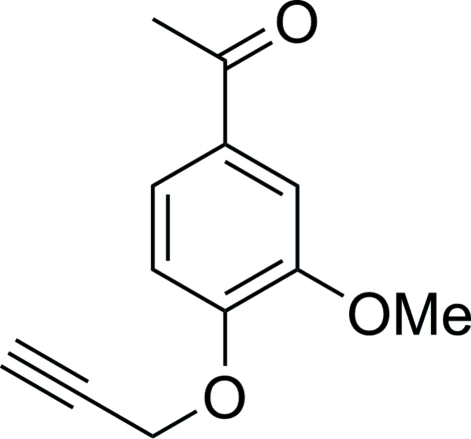


## Experimental

### 

#### Crystal data

C_12_H_12_O_3_
*M* *_r_* = 204.22
Monoclinic, 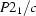

*a* = 12.152 (2) Å
*b* = 8.9870 (18) Å
*c* = 10.179 (2) Å
β = 103.86 (3)°
*V* = 1079.3 (4) Å^3^
*Z* = 4
Mo *K*α radiation
μ = 0.09 mm^−1^
*T* = 293 K
0.30 × 0.20 × 0.10 mm

#### Data collection

Enraf–Nonius CAD-4 diffractometer
Absorption correction: ψ scan (North *et al.*, 1968 ▶) *T*_min_ = 0.974, *T*_max_ = 0.991
2908 measured reflections
1988 independent reflections
1400 reflections with *I* > 2σ(*I*)
*R*_int_ = 0.052
3 standard reflections every 200 reflections  intensity decay: 1%

#### Refinement

*R*[*F* ^2^ > 2σ(*F* ^2^)] = 0.056
*wR*(*F* ^2^) = 0.169
*S* = 1.00
1988 reflections
141 parameters
H atoms treated by a mixture of independent and constrained refinement
Δρ_max_ = 0.21 e Å^−3^
Δρ_min_ = −0.20 e Å^−3^

### 

Data collection: *CAD-4 EXPRESS* (Enraf–Nonius, 1994 ▶); cell refinement: *CAD-4 EXPRESS*; data reduction: *XCAD4* (Harms & Wocadlo, 1996 ▶); program(s) used to solve structure: *SHELXS97* (Sheldrick, 2008 ▶); program(s) used to refine structure: *SHELXL97* (Sheldrick, 2008 ▶); molecular graphics: *SHELXTL* (Sheldrick, 2008 ▶); software used to prepare material for publication: *SHELXTL*.

## Supplementary Material

Crystal structure: contains datablocks I, global. DOI: 10.1107/S1600536810052074/nc2210sup1.cif

Structure factors: contains datablocks I. DOI: 10.1107/S1600536810052074/nc2210Isup2.hkl

Additional supplementary materials:  crystallographic information; 3D view; checkCIF report

## Figures and Tables

**Table 1:** Hydrogen-bond geometry (Å, °)

*D*—H⋯*A*	*D*—H	H⋯*A*	*D*⋯*A*	*D*—H⋯*A*
C12—H12*A*⋯O2^i^	0.90 (4)	2.40 (4)	3.270 (3)	164 (3)

Symmetry code: (i) 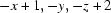.

**Fig. 1.**
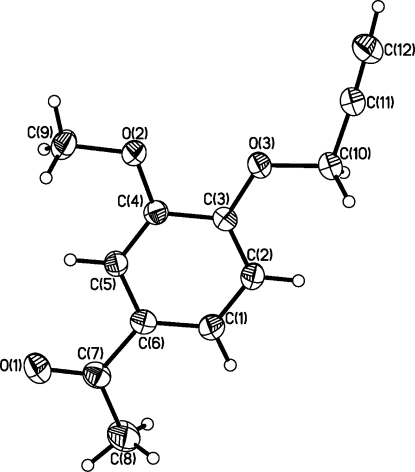
Molecular structure of the title compound, showing the atom-numbering scheme. Displacement ellipsoids are drawn at the 50% probability level.

## supplementary crystallographic information

### Comment

Lignin is natural polymer occurring in plant cell walls and considered to be the second most abundant biopolymer after cellulose and the β-O-4 structure is the most abundant substructure in lignin (Cathala B. *et al.*, 2003). Lignin is an amorphous polyphenolic material arising from an enzyme-mediated dehydrogenate polymerization of three major phenylpropanoid monomers, i. e., coniferyl, sinapyl and p-coumaril alcohol. Therefore, lignin can be oxidized to produce syringaldehyde, vanillin, *p*-hydroxybenzaldehyde and acetovanillone etc. Acetovanillone and vanillin are usually used to synthesize lignin mimics (Kishimoto T. *et al.*, 2005). In order to prepare well defined linear lignin mimics composed of the β-O-4 structure by "Click Chemistry" using acetovanillone, an intermediate product C_12_H_12_O_3_, the title compound was synthesized and identified by crystal structure analysis. In the molecular structure of the title compound, the acetophenone unit is almost a planar with a torsion angle C5—C6—C7—O1, -3.5 (3)° (Fig. 1). In addition, the methoxy group and the prop-2-ynyloxy group are nearly coplanar with the attached benzene ring [C9—O2—C4—C5 = 1.2 (3)° and C10—O3—C3—C2, 2.2 (3)°]. In the crystal structure weak intermolecular C_terminal alkynes_—H···O_methoxy_ interactions aref found.

### Experimental

A mixture of 4'-hydroxy-3'-methoxyacetophenon (5 mmol), propargyl bromide (5 mmol) and triethylamine (5 mmol) was stirred in acetone (20 ml) at 353 K. After completion of the reaction (TLC monitoring), the reaction mixture was diluted with ether (100 ml) and washed with water 3 times. The organic phase was dried over with anhydrous Na_2_SO_4_ and concentrated to dryness in *vacuo*. The obtained crude crystalline was purified by column chromatography to obtain a pure white solid. Colourless single crystals suitable for X-ray crystallographic analysis were grown by slow evaporation of an ethyl actate solution of the title compound.

### Refinement

The H atoms were fixed geometrically and allowed to ride on the attached non-H atoms, with C—H = 0.93–0.97 Å, and with *U*_iso_(H) = 1.5 *U*_eq_(C) for methyl H atoms and 1.2 *U*_eq_(C) for all other atoms.

### Crystal data

**Table:**

C_12_H_12_O_3_	*F*(000) = 432
*M**_r_* = 204.22	*D*_x_ = 1.257 Mg m^−^^3^
Monoclinic, *P*2_1_/*c*	Mo *K*α radiation, λ = 0.71073 Å
Hall symbol: -P 2ybc	Cell parameters from 25 reflections
*a* = 12.152 (2) Å	θ = 9–13°
*b* = 8.9870 (18) Å	µ = 0.09 mm^−^^1^
*c* = 10.179 (2) Å	*T* = 293 K
β = 103.86 (3)°	Block, colourless
*V* = 1079.3 (4) Å^3^	0.30 × 0.20 × 0.10 mm
*Z* = 4	

### Data collection

**Table:**

Enraf–Nonius CAD-4 diffractometer	1400 reflections with *I* > 2σ(*I*)
Radiation source: fine-focus sealed tube	*R*_int_ = 0.052
graphite	θ_max_ = 25.4°, θ_min_ = 1.7°
ω/2θ scans	*h* = −14→0
Absorption correction: ψ scan (North *et al.*, 1968)	*k* = −3→10
*T*_min_ = 0.974, *T*_max_ = 0.991	*l* = −11→12
2908 measured reflections	3 standard reflections every 200 reflections
1988 independent reflections	intensity decay: 1%

### Refinement

**Table:**

Refinement on *F*^2^	Secondary atom site location: difference Fourier map
Least-squares matrix: full	Hydrogen site location: inferred from neighbouring sites
*R*[*F*^2^ > 2σ(*F*^2^)] = 0.056	H atoms treated by a mixture of independent and constrained refinement
*wR*(*F*^2^) = 0.169	*w* = 1/[σ^2^(*F*_o_^2^) + (0.1*P*)^2^ + 0.1*P*] where *P* = (*F*_o_^2^ + 2*F*_c_^2^)/3
*S* = 1.00	(Δ/σ)_max_ < 0.001
1988 reflections	Δρ_max_ = 0.21 e Å^−^^3^
141 parameters	Δρ_min_ = −0.20 e Å^−^^3^
0 restraints	Extinction correction: *SHELXL97* (Sheldrick, 2008), Fc^*^=kFc[1+0.001xFc^2^λ^3^/sin(2θ)]^-1/4^
Primary atom site location: structure-invariant direct methods	Extinction coefficient: 0.066 (9)

### Special details

**Table:**

Experimental. Absorption correction: semi-empirical absorption based on psi-scan (North *et al.*, 1968)
Geometry. All esds (except the esd in the dihedral angle between two l.s. planes) are estimated using the full covariance matrix. The cell esds are taken into account individually in the estimation of esds in distances, angles and torsion angles; correlations between esds in cell parameters are only used when they are defined by crystal symmetry. An approximate (isotropic) treatment of cell esds is used for estimating esds involving l.s. planes.
Refinement. Refinement of *F*^2^ against ALL reflections. The weighted *R*-factor *wR* and goodness of fit *S* are based on *F*^2^, conventional *R*-factors *R* are based on *F*, with *F* set to zero for negative *F*^2^. The threshold expression of *F*^2^ > σ(*F*^2^) is used only for calculating *R*-factors(gt) *etc*. and is not relevant to the choice of reflections for refinement. *R*-factors based on *F*^2^ are statistically about twice as large as those based on *F*, and *R*- factors based on ALL data will be even larger.

### Fractional atomic coordinates and isotropic or equivalent isotropic displacement parameters (Å^2^)

**Table:**

	*x*	*y*	*z*	*U*_iso_*/*U*_eq_	
O1	0.02729 (15)	−0.0061 (2)	0.20836 (17)	0.0658 (6)	
C1	0.29554 (18)	0.1655 (3)	0.3643 (2)	0.0511 (6)	
H1A	0.3039	0.2296	0.2956	0.061*	
O2	0.26138 (12)	−0.10284 (18)	0.68203 (14)	0.0529 (5)	
C2	0.37931 (19)	0.1593 (3)	0.4840 (2)	0.0506 (6)	
H2A	0.4444	0.2170	0.4943	0.061*	
O3	0.44025 (13)	0.05304 (19)	0.71063 (15)	0.0527 (5)	
C3	0.36568 (17)	0.0674 (2)	0.5876 (2)	0.0428 (5)	
C4	0.26800 (18)	−0.0201 (2)	0.5718 (2)	0.0409 (5)	
C5	0.18711 (18)	−0.0157 (2)	0.4518 (2)	0.0426 (6)	
H5A	0.1230	−0.0754	0.4405	0.051*	
C6	0.19980 (18)	0.0773 (2)	0.3462 (2)	0.0433 (6)	
C7	0.1073 (2)	0.0778 (3)	0.2195 (2)	0.0493 (6)	
C8	0.1143 (2)	0.1817 (4)	0.1077 (3)	0.0835 (10)	
H8A	0.0491	0.1684	0.0338	0.125*	
H8B	0.1818	0.1612	0.0775	0.125*	
H8C	0.1164	0.2824	0.1397	0.125*	
C9	0.1630 (2)	−0.1939 (3)	0.6694 (3)	0.0616 (7)	
H9A	0.1675	−0.2465	0.7526	0.092*	
H9B	0.1590	−0.2640	0.5973	0.092*	
H9C	0.0965	−0.1323	0.6498	0.092*	
C10	0.54011 (18)	0.1439 (3)	0.7385 (2)	0.0510 (6)	
H10A	0.5201	0.2485	0.7291	0.061*	
H10B	0.5873	0.1203	0.6768	0.061*	
C11	0.59931 (19)	0.1100 (3)	0.8775 (3)	0.0547 (6)	
C12	0.6418 (3)	0.0809 (4)	0.9899 (3)	0.0737 (9)	
H12A	0.669 (3)	0.067 (4)	1.079 (4)	0.094 (11)*	

### Atomic displacement parameters (Å^2^)

**Table:**

	*U*^11^	*U*^22^	*U*^33^	*U*^12^	*U*^13^	*U*^23^
O1	0.0531 (10)	0.0781 (13)	0.0590 (11)	−0.0090 (10)	−0.0009 (8)	0.0004 (9)
C1	0.0513 (13)	0.0543 (14)	0.0470 (12)	−0.0053 (12)	0.0105 (10)	0.0068 (11)
O2	0.0519 (9)	0.0597 (10)	0.0453 (9)	−0.0136 (8)	0.0080 (7)	0.0078 (7)
C2	0.0457 (12)	0.0544 (14)	0.0507 (13)	−0.0109 (11)	0.0097 (10)	0.0047 (11)
O3	0.0472 (9)	0.0611 (10)	0.0448 (9)	−0.0132 (8)	0.0011 (7)	0.0045 (7)
C3	0.0422 (11)	0.0469 (13)	0.0377 (11)	−0.0025 (10)	0.0065 (9)	−0.0021 (10)
C4	0.0446 (11)	0.0379 (11)	0.0411 (11)	−0.0014 (9)	0.0120 (9)	−0.0010 (9)
C5	0.0397 (11)	0.0439 (12)	0.0443 (12)	−0.0027 (10)	0.0105 (9)	−0.0046 (10)
C6	0.0456 (12)	0.0429 (12)	0.0411 (11)	0.0044 (10)	0.0096 (9)	−0.0007 (9)
C7	0.0467 (12)	0.0523 (14)	0.0464 (13)	0.0077 (12)	0.0065 (10)	−0.0001 (11)
C8	0.0727 (18)	0.102 (2)	0.0613 (16)	−0.0064 (18)	−0.0130 (14)	0.0324 (17)
C9	0.0553 (14)	0.0652 (16)	0.0652 (15)	−0.0146 (13)	0.0160 (12)	0.0131 (13)
C10	0.0437 (12)	0.0570 (14)	0.0495 (13)	−0.0083 (11)	0.0056 (10)	−0.0030 (11)
C11	0.0470 (12)	0.0611 (16)	0.0533 (14)	−0.0080 (12)	0.0071 (11)	−0.0022 (12)
C12	0.0720 (18)	0.086 (2)	0.0551 (17)	−0.0111 (16)	−0.0008 (14)	0.0035 (16)

### Geometric parameters (Å, °)

**Table:**

O1—C7	1.214 (3)	C6—C7	1.494 (3)
C1—C6	1.383 (3)	C7—C8	1.490 (4)
C1—C2	1.389 (3)	C8—H8A	0.9600
C1—H1A	0.9300	C8—H8B	0.9600
O2—C4	1.365 (3)	C8—H8C	0.9600
O2—C9	1.429 (3)	C9—H9A	0.9600
C2—C3	1.381 (3)	C9—H9B	0.9600
C2—H2A	0.9300	C9—H9C	0.9600
O3—C3	1.365 (3)	C10—C11	1.457 (3)
O3—C10	1.433 (3)	C10—H10A	0.9700
C3—C4	1.400 (3)	C10—H10B	0.9700
C4—C5	1.373 (3)	C11—C12	1.167 (4)
C5—C6	1.399 (3)	C12—H12A	0.90 (3)
C5—H5A	0.9300		
			
C6—C1—C2	120.7 (2)	C8—C7—C6	119.5 (2)
C6—C1—H1A	119.7	C7—C8—H8A	109.5
C2—C1—H1A	119.7	C7—C8—H8B	109.5
C4—O2—C9	116.81 (17)	H8A—C8—H8B	109.5
C3—C2—C1	119.8 (2)	C7—C8—H8C	109.5
C3—C2—H2A	120.1	H8A—C8—H8C	109.5
C1—C2—H2A	120.1	H8B—C8—H8C	109.5
C3—O3—C10	118.19 (17)	O2—C9—H9A	109.5
O3—C3—C2	125.65 (19)	O2—C9—H9B	109.5
O3—C3—C4	114.25 (18)	H9A—C9—H9B	109.5
C2—C3—C4	120.1 (2)	O2—C9—H9C	109.5
O2—C4—C5	125.21 (19)	H9A—C9—H9C	109.5
O2—C4—C3	115.26 (19)	H9B—C9—H9C	109.5
C5—C4—C3	119.53 (19)	O3—C10—C11	105.73 (19)
C4—C5—C6	120.9 (2)	O3—C10—H10A	110.6
C4—C5—H5A	119.6	C11—C10—H10A	110.6
C6—C5—H5A	119.6	O3—C10—H10B	110.6
C1—C6—C5	119.0 (2)	C11—C10—H10B	110.6
C1—C6—C7	123.2 (2)	H10A—C10—H10B	108.7
C5—C6—C7	117.8 (2)	C12—C11—C10	176.8 (3)
O1—C7—C8	120.6 (2)	C11—C12—H12A	173 (2)
O1—C7—C6	119.9 (2)		
			
C6—C1—C2—C3	−1.8 (4)	C3—C4—C5—C6	−1.4 (3)
C10—O3—C3—C2	2.2 (3)	C2—C1—C6—C5	1.6 (3)
C10—O3—C3—C4	−176.96 (19)	C2—C1—C6—C7	−179.4 (2)
C1—C2—C3—O3	−178.8 (2)	C4—C5—C6—C1	0.0 (3)
C1—C2—C3—C4	0.4 (3)	C4—C5—C6—C7	−179.01 (19)
C9—O2—C4—C5	1.2 (3)	C1—C6—C7—O1	177.5 (2)
C9—O2—C4—C3	−179.78 (19)	C5—C6—C7—O1	−3.5 (3)
O3—C3—C4—O2	1.4 (3)	C1—C6—C7—C8	−2.5 (4)
C2—C3—C4—O2	−177.8 (2)	C5—C6—C7—C8	176.5 (2)
O3—C3—C4—C5	−179.54 (19)	C3—O3—C10—C11	177.2 (2)
C2—C3—C4—C5	1.2 (3)	O3—C10—C11—C12	−25 (6)
O2—C4—C5—C6	177.52 (19)		

### Hydrogen-bond geometry (Å, °)

**Table:**

*D*—H···*A*	*D*—H	H···*A*	*D*···*A*	*D*—H···*A*
C12—H12A···O2^i^	0.90 (4)	2.40 (4)	3.270 (3)	164 (3)

Symmetry codes: (i) −*x*+1, −*y*, −*z*+2.
